# Cancer-Associated Fibroblasts in Conversation with Tumor Cells in Endometrial Cancers: A Partner in Crime

**DOI:** 10.3390/ijms22179121

**Published:** 2021-08-24

**Authors:** De Pradip, Aske Jennifer, Dey Nandini

**Affiliations:** Translational Oncology Laboratory, Avera Cancer Institute, Sioux Falls, SD 57105, USA; Pradip.De@avera.org (D.P.); Jennifer.Aske@avera.org (A.J.)

**Keywords:** tumor micro-environment, cancer-associated fibroblasts, metastasis-associated phenotypes, immune-defense, stromagenesis

## Abstract

A tumor cell carrying characteristic genomic alteration(s) exists within its host’s microenvironment. The tumor microenvironment (TME) renders holistic support to the tumor via cross-talk between tumor cells and three components of TME, immune components, vascular components, and fibroblast components. The tempero-spatial interaction of tumor cells with its microenvironment is the deterministic factor for tumor growth, progression, resistance to therapy, and its outcome in clinics. TME (1) facilitates proliferation, and the ensuing metastasis-associated phenotypes, (2) perturbs immune surveillance and supports tumor cells in their effort to evade immune recognition, and (3) actively participates in developing drug-induced resistance in cancer cells. Cancer-Associated Fibroblast (CAF) is a unique component of TME. CAF is the host mesenchyme immediately surrounding the tumor cells in solid tumors. It facilitates tumor growth and progression and participates in developing drug resistance in tumor cells by playing a critical role in all the ways mentioned above. The clinical outcome of a disease is thus critically contributed to by the CAF component of TME. Although CAFs have been identified historically, the functional relevance of CAF-tumor cell cross-talk and their influence on angiogenic and immune-components of TME are yet to be characterized in solid tumors, especially in endometrial cancers. Currently, the standard of care for the treatment of endometrial cancers is primarily guided by therapies directed towards the disease’s tumor compartment and immune compartments. Unfortunately, in the current state of therapies, a complete response (CR) to the therapy is still limited despite a more commonly achieved partial response (PR) and stable disease (SD) in patients. Acknowledging the limitations of the current sets of therapies based on only the tumor and immune compartments of the disease, we sought to put forward this review based on the importance of the cross-talk between CAF of the tumor microenvironment and tumor cells. The premise of the review is to recognize the critical role of CAF in disease progression. This manuscript presents a systemic review of the role of CAF in endometrial cancers. We critically interrogated the active involvement of CAF in the tumor compartment of endometrial cancers. Here we present the functional characteristics of CAF in the context of endometrial cancers. We review (1) the characteristics of CAF, (2) their evolution from being anti-tumor to pro-tumor, (3) their involvement in regulating growth and several metastasis-associated phenotypes of tumor cells, (4) their participation in perturbing immune defense and evading immune surveillance, and (5) their role in mediating drug resistance via tumor-CAF cross-talk with particular reference to endometrial cancers. We interrogate the functional characteristics of CAF in the light of its dialogue with tumor cells and other components of TME towards developing a CAF-based strategy for precision therapy to supplement tumor-based therapy. The purpose of the review is to present a new vision and initiate a thought process which recognizes the importance of CAF in a tumor, thereby resulting in a novel approach to the design and management of the disease in endometrial cancers.

## 1. Definition of CAF

Cancer-associated fibroblasts (CAFs) in solid tumors can be defined as a dynamically plastic host mesenchymal fibroblastic component of TME (tumor microenvironment) immediately surrounding the tumor cells. CAFs are a heterogeneous population of cells with extraordinarily numerous cells of origin and modes of activation [1,2,3,4,5,6,7,8,9]. CAFs are resident fibroblasts and other mesenchymal components of the host, which are transformed/activated by the tumor cell. CAF presents a quadruple-negative status. Based on lineage exclusion, CAFs are negative for (1) epithelial markers, (2) endothelial markers, (3) leukocyte markers, and (4) mutations found within cancer cells [9]. On the other hand, based on positivity for the mesenchymal marker(s) closely similar to normal fibroblasts, CAFs are positive for alpha-SMA (ACTA2), vimentin, FAP-1, FSP-1(fibroblast-specific protein-1; S100A4), CD90 (Thy-1), Tie-7, and PDGFR in different subsets. The markers of CAFs are reviewed elsewhere [8,9]. These markers make CAF similar to tissue-resident normal fibroblasts participating in very similar functions like normal fibroblasts, including matrix remodeling, immune-cross talk (via T-cell/macrophages/endothelium), secretory functions, inflammation, and tissue remodeling, which supports healthy tissue. Yet, CAFs in their activated forms are far from supportive of healthy tissue. CAFs, on the contrary, support tumor cells metabolically and immunologically via their role in (1) “stromagenesis”, (2) immune-escape, (3) metastasis-associated phenotypes/(Epithelial-MesenchymalTransition) EMT, (4) growth-promoting secretory function, (5) development of drug-resistance, (6) angiogenesis, and (7) stemness [10,11,12]. Thus, functions of CAFs are as diverse as their cells of origin, modes of origin, markers, and sub-populations/specialization [2,9,13,14,15,16,17].

## 2. CAF as an Evolving Component of Tumor Microenvironment 

**Figure 1:**
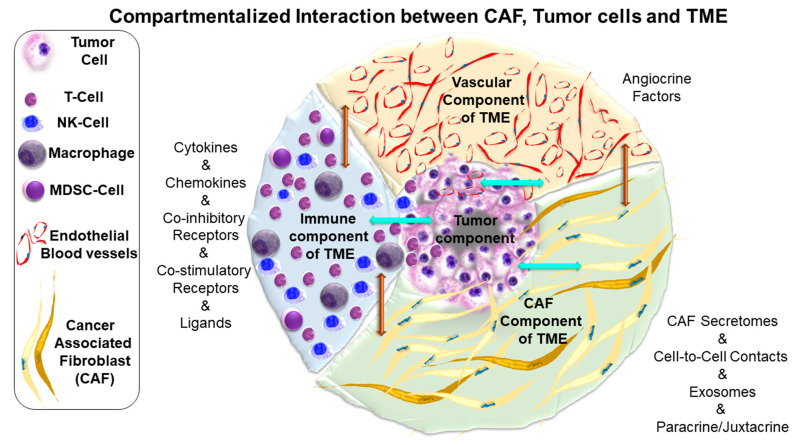
Diagrammatic representation of the compartmentalized relationship of cancer-associated fibroblasts (CAF) with tumor cells and other components of the tumor microenvironment (TME): CAF interacts and has a bidirectional influence on other two major components of TME, including the immune component and vascular component (vertical brown arrows). All three components of TME interact (vertical brown arrows) and have bidirectional interaction with the tumor compartment (horizontal cyan arrows). The bidirectional interactions are not compartmentalized in space as all the components are in close proximity for autocrine/paracrine interactions but compartmentalized on a cell-to-cell basis. CAFs have distinctly compartmentalized bidirectional interactions with cells of the immune component and vascular component of TME, which are distinct from the bidirectional interactions with tumor cells. These characteristic interactions are (1) via secretomes, (2) autocrine, paracrine, and angiocrine in nature, and/or (3) cell-to-cell interactions. Interestingly, the bidirectional interactions are also in real-time. For example, CAF’s interaction with other components of TME and tumor cells changes following treatment of the tumor with drug(s), which may be functionally connected to the development of resistance. Schematically, each component of TME is compartmentalized, and a few cells of each component are presented within the tumor compartment. Heterogeneity of CAF is represented in two shades of color.

As presented above, CAF is a component of TME. TME is the niche that provides metabolic support, immune surveillance, angiocrine, and inflammatory milieu to the tumor cells in a host’s body. The TME is the deterministic and dynamic context of a solid tumor [18]. The TME is comprised of three interactive components: the mesenchymal component, immune component, and vascular component. All three components of TME interact with and influence each other in synchrony with tumor cells. Tumors evolve. CAF, in interacting with the rest of the components of TME, co-evolves with the tumor in supporting the progression of the tumor (Figure 1). Tumor cells bearing a characteristic genomic background cross-talk with their TME in favor of their proliferation and metastasis-associated phenotypes at a particular stage of tumor growth. Such an evolving interaction is established via TME-tumor cross-talk. TME-tumor cross-talk and its evolution as a tumor progresses are critical events in regulating the tumor’s fate, thus affecting the outcome of the management of a disease. Hence, tumorigenesis, when well supported by CAF, exhibits a higher probability of developing a progressive disease and drug resistance in response to treatment [9,19].

## 3. CAF & CAF Conundrum

CAF is a unique component of TME because it bears a strong element of ambiguity. CAFs present riddles; beginning with their definition, origin, markers, and functions in the context of tumorigenesis, tumor progression, and drug resistance. First, CAFs are characteristically different from the normal tissue-resident fibroblasts and yet retain their cardinal elongated fibroblastic-morphology, mesenchymal-markers, and major fibroblastic functions in a modified way [20].

Secondly, CAF cross-talks with all components of TME as well as tumor cells and thus bears a strong potential to become a target cell to contribute to the novel CAF-targeted treatment strategies [2,21,22,23]. Yet, CAFs are the least characterized, the least understood, and the most underutilized component of a tumor so far to aid the management of the disease. Although CAFs are the most abundantly present, easily cultured/modeled, and experimentally manipulated critical elements of TME, their origin, markers, subpopulation, and functions remain largely inconclusive in many solid tumors. 

Third, within the TME of an advanced stage tumor, CAFs support tumor growth and metastasis of the tumor cells and co-evolve with the tumor progression and development of drug resistance. Yet CAF-directed management of solid tumors is minimal owing to the well-known multifaceted nature and function of CAFs [2,24]. This puzzling dichotomy is the single most characteristic nature of CAF, which makes them intriguing yet of limited applicability in the treatment of cancers. The Banbury Center meeting was convened to state the current understanding of the biology of CAFs, their origin, fundamental properties, and delineate their role for medical applications in targeting cancers [9]. 

## 4. CAF in Endometrial Cancers

Compared to solid tumors of other organ types, uterine endometrium presents an abundance of fibroblast-enriched stroma embedding the glandular epithelium. At a certain point of their oncogenic progression, endometrial cancer cells orchestrate the transformation of normal residential fibroblasts in the stroma into CAFs. In the TME of endometrial cancers, CAFs acquire cancer-specific characteristics in addition to their primarily fibroblastic background. Myofibroblast-rich cell populations constituting the tumor stroma associated with the host’s immediate extra-tumoral cellular elements are designated as CAFs. However, CAFs have also been reported to originate from non-myofibroblastic cells [2,8]. Various modes of CAF activation are reviewed elsewhere [9,25,26]. Modification of normal residential fibroblasts to activated/transdifferentiated myofibroblasts (a type of CAF) infiltrating carcinoma is irreversible as, once activated, CAFs preserve their pro-tumor property even in the absence of direct contact with tumor cells in vitro in many solid tumors [27,28,29]. Depending on the stages of neoplasia, tumor cells evolve due to their accumulation of many genetic changes, chromosomal aberrations, epigenetic changes, and they influence the co-evolution of all the components of TME, including CAFs [30]. The transformation from residential fibroblasts to CAF is a non-genomic alteration-mediated event in TME towards cancer progression. Thus CAFs play a critical role in “turning the table” in favor of tumor cells towards the progression and metastasis of the disease in patients, leading to a higher grade of malignancy and poor prognosis [31,32]. 

CAFs are the single most critical deterministic component of TME in contributing to aggressiveness and drug resistance. Overall, the property and function of CAFs are directly linked to their origin in a specific organ-type cancer(s) and the history of the tumor progression, and the response to drug treatment. The leading role of CAF of TME in the progression and the distance dissemination of cancer cells in an immune-safe, growth-supporting, and metastasis-promoting stromal milieu is the outcome of a dynamic cross-talk between CAF and tumor cells in time and space (Figure 1). In the course of the real-time evolution of tumor cells, there occurs a rate-limiting conversion of CAFs in TME from pro-tumor state to anti-tumor state, “stromagenesis”. In an established CAF-driven endometrial tumor, a cross-talk between CAF and tumor cells, the stromal milieu eventually acquires specific properties leading to a vicious circle of an incredibility complicated signaling choreograph among tumor cells, fibroblasts, pericytes, lymphocytes, endothelial cells, and tumor associated-macrophages. Thus, the mechanism of reverse conversion of CAFs from pro-tumor state to anti-tumor state will be essential to the construction of a disease management strategy based on targeting the reversal of “stromagenesis”, stromal-switch, or normalization of stroma from pro-tumor to anti-tumor state. Signaling pathways involved in the cross-talk between cancer cells and stromal CAF in gynecological malignancies, including endometrial cancers, may have therapeutic implications because of the role of the estrogen-mediated release of chemokines and cytokines [33]. CAF in the TME has a high therapeutic potential, offering many targeted and immunological therapies. In endometrial cancers, such clinical management of the disease based on targeting “stromagenesis” remains elusive at the present time due to the paucity of conclusive data to support the origin, subpopulation, heterogeneity, and definitive functions of CAF.

## 5. Characteristics of CAF-Tumor Cross-Talk 

The functional relationship between CAF and tumor cells is one of the best examples of the TME-tumor cross talk. The cross-talk of CAF with tumor cells is the building block of the role of CAF. The CAF-tumor dialogue mediates all functions of CAF and thus controls the fate of tumor cells in real-time. The understanding of the cross-talk will provide insight into the future of stromal-based targeting to manage solid tumors, which bears a dominant CAF component. Like other solid tumors, the cross-talk between CAF and tumor cells has been reported in gynecological malignancies, including uterine cancers, highlighting the high translational relevance of a CAF-based therapy in these organ-type cancers [33]. A genetically transformed tumor cell evolves its functions and relationships with stromal cells, including residential fibroblasts, which eventually get activated into CAFs. Cytokine secretion (CAF secretomes), cell-to-cell contacts (via co-stimulatory and co-inhibitor molecules), and exosomes are the language of this complex paracrine/juxtacrine intratumoral dialogue, which involves all potential phenotypes of a tumor cell, including tumor growth, angiogenesis, metabolic reprogramming, immune evasion, immune suppression, metastasis, and chemoresistance [8,34,35].

The relationship between CAF and tumor growth is both bi-phasic and autocrine/paracrine, depending on the stage, grade, and tumor type. CAFs are tumoricidal at the initial stage of tumor growth, as reported in pancreatic ductal adenocarcinoma (PDAC) [11]. The CAF-tumor cross-talk is also bi-directional, dynamic, multi-nodal and involves: (1) transcriptional activation/repression of oncogenic factors, (2) the regulation of different oncogenic pathways at the miRNA or protein levels, and (3) the activation/suppression of cells belonging to the tumor compartment and other components of TME, such as the immune compartment or angiogenic compartment. The cross-talk between CAF and tumor cells can be stratified in two ways: (1) the modes of the cross-talk and (2) the tumor cell functions affected by the cross-talk. There can be several modes and mediators of the cross-talk in endometrial tumors, which can affect a number of cellular functions to control many phenotypes in the endometrial tumor cells. 

The bi-directional cross-talk between CAF and tumor cells involves putative secretory signaling proteins such as TGF-beta 2, FGF2 (from CAF), and FGFR1 (from tumor cells). Interestingly PD-L1 expressed in CAF interacts with its cognate receptor, PD-1 expressed in the surrounding T-cells, one of the established cell-to-cell mechanisms of immune evasion. Although the cross-talk between CAFs and well-adapted tumor cells in other solid tumors has been identified historically [8,34,35,36,37], the functional relevance of the cross-talk and their influence on angiogenic and immune components of TME in endometrial cancers are yet to be fully characterized. 

## 6. Language and Topic of Cross-Talk between CAF and Tumor Cells in Endometrial Cancers 

Tumor cells are engaged to CAF via the paracrine/juxtacrine mode and vice versa [38]. The mode of signal transduction between CAF and tumor cells has been referred to as the “Language of Cross-Talk”, and how the cross-talk is involved or initiates different functions in the tumor cell has been referred to as the “Agenda of Cross-Talk” as presented in Table 1. The agendas of the cross-talk are: (1) growth, (2) proliferation, (3) metastasis-associated phenotypes, (4) metabolic reprogramming, and (5) immunological reprogramming. Table 1 shows that most studies have been conducted on the effect of the CAF-tumor cell interaction in mediating the growth and proliferation of endometrial tumor cells. Interestingly, most of the CAF-mediated growth involved estrogen signaling via CAF secretome. In contrast, functions like metastasis-associated phenotypes, EMT, progression, and stemness were mediated via transcriptional regulation of genes, exosomes, and miRNAs. Although the studies on metabolic and immunologic reprogramming are limited in the current time in endometrial cancers, future work will identify the precise nature of this cross-talk. The details of CAF’s influence on different features of endometrial tumor cells have been presented below.

## 7. CAF Influencing Proliferation and Growth of Tumor Cells in Endometrial Cancers 

### 7.1. Steroid-Driven Proliferation of Endometrial Tumor Cells

In uterine cancers, steroid-driven cell growth has been the primary mode of the proliferation of endometrial tumor cells. The effect of endometrial-CAFs on hormone-driven responses in endometrial cancers provides an opportunity to study the effect of CAF on steroid receptor signaling. The characteristic tissue architecture of the endometrium includes the proximity of its stromal population(s) to epithelial glands. The endometrial CAF-tumor cell cross-talk which mediates the proliferation of tumor cells is intimately wired to the age of the uterus and its steroidal milieu. The stromal cells are known to orchestrate the differentiation and the proliferation of the near epithelium components via critical juxtacrine and paracrine activity of estrogen receptor (ESR1), which mediates the release of various growth factors and cell-cycle-related proteins. The juxtacrine action of ER-alpha in uterine stromal cells is needed for estrogen-mediated epithelial cell proliferation [39]. A very early report on the influence and interaction between tumor cells and endometrial fibroblast came from a study by Imai et al. [40]. Extracts from uterine and cervical cancers exhibited a growth-promoting activity and induced proliferation of human endometrial fibroblasts. The hormonal influence can be enhanced by the oncogenic alterations of E-cadherins, the Wnt pathway leading to EMT phenotypes which involves a close signaling network with mesenchymal fibroblasts and myofibroblasts. Intra-tumoral, mesenchymal, and tumor-mesenchymal interaction of sex steroids, Wnt pathway proteins, and EMT-associated proteins are the elements of cross-talk which constitute the “stromal clues” in endometrial cancers [41]. Peri-tumoral CAFs are ERα+ fibroblasts and myofibroblasts are activated by the binding of 17-beta-estradiol and its stromal receptor ER-alpha. They secrete cell-cycle-related proteins (MAD2L1, CDKN1A, CEBPbeta) and growth factors (IGF, TGF), inducing paracrine effect leading to migration, invasion, evasion of apoptosis, and EMT in the epithelial tumor cells [33]. Estrogen and progesterone exhibited signature effects on fibroblasts and tumor cell lines. Estrogen has been reported to increase c-fos expression and protein kinase C (PKC) activity in fibroblasts and Ishikawa cells but not in HHUA cells. In comparison, progesterone diminished c-fos and c-jun expression and PKC activity induced by estradiol in the fibroblasts, but not in Ishikawa cells, which persistently overexpressed c-fos and c-jun [42]. Estrogen has been shown to induce c-HRas expression via activation of tyrosine kinase in uterine endometrial fibroblasts and cancer cells, wherein estrogen increased c-HRas expression and tyrosine kinase activity in both fibroblast and Ishikawa cells [43,44]. Pineda et al. studied how normal and cancer-associated stromal cells from patients with and without endometrial cancer affected endometrial tumor growth in response to estrogen and progesterone. Their study showed that endometrial-cancer-associated cells responded differently to in vitro hormone treatment than benign endometrial stromal cells. The presence of both benign and high-grade cancer-associated stromal cells increased estrogen-driven tumor growth in their xenograft models. Progesterone attenuated tumor growth in Ishikawa plus benign or high-grade stromal cells, but not in Ishikawa cells alone or with low-grade stromal cells [45]. 

### 7.2. Non-Steroidal Proliferation of Endometrial Tumor Cells 

Classical oncogenic signals have been identified as mediating the proliferation of tumor cells in response to the paracrine influence of CAF. Conditioned media from the CAF primary cultures from endometrial cancer tissues dose-dependently increased proliferation of both primary cultures and cell lines of endometrial cancer in vitro when compared to non-treated cells, in contrast to those from normal endometrial fibroblast cell line (51%) (*p* < 0.0001) [46]. The increase in proliferation involved both the PI3K/AKT and MAPK/ERK pathways in endometrial cancer cells. 

Other classical oncogenic pathways have been reported to be involved in the non-paracrine steroidal proliferation of endometrial tumor cells under the secretory paracrine influence of pro-tumorigenic CAF. Subramaniam et al. demonstrated that isolated endometrial CAFs secrete interleukin-6 (IL-6) which induces proliferation of cancer cells via c-Myc expression mediated STAT-3 transcriptional activity [47].

The uterus is an organ that undergoes changes in three ways in a lifetime of a woman, pubertal change (lunar cycles), changes during pregnancy/postpartum, and post-menopausal aging. Interesting studies have demonstrated age-related changes in the stromal-epithelial cell interactions that facilitate tumorigenesis in the uterus [48,49]. Rinehart et al. demonstrated that aging human endometrial stromal fibroblasts reverses their influence over endometrial epithelial cells. Adult human endometrial stromal fibroblasts were found to block tumorigenesis (inhibit anchorage-independent proliferation, restrain colony outgrowth, and induce normal tissue architecture formation). As stromal fibroblasts age, these inhibitory influences on tumor-associated phenotypes are reversed and become stimulatory. Age-related changes in interleukin-1alpha and pigment epithelium-derived factor/early population doubling cDNA-1 expression were found to be the molecular determinant of interactive senescence and interaction between stromal fibroblasts and epithelial cells in the uterus.

## 8. CAF Influencing Metastasis-Associated Phenotypes of Tumor Cells in Endometrial Cancers

### 8.1. Matrix Organization & Stromal Architecture

Being mostly fibroblast in origin, CAFs actively participate in the matrix organization and remodel the stromal architecture, known as stromagenesis. The stroma acts as a floor that controls the movement of tumor cells (integrin-directed cell movement) as a part of many metastasis-associated phenotypes, including adhesion, migration, invasion, and EMT. The physico-chemical properties of stroma closely influence the metastasis-associated phenotypes of tumor cells. CAFs induce a collagen cross-link switch. Lower lysine-aldehyde-derived collagen cross-links to hydroxylysine aldehyde-derived collagen. It has been reported that cross-links switch in stroma induced by CAFs promote the migratory and invasive properties of tumor cells in lung adenocarcinomas [50]. CAFs enhanced the invasive properties of tumor cells in 3D collagen gels. Lysyl hydroxylase 2 (PLOD2/LH2), which drives hydroxylysine aldehyde-derived collagen cross-links formation, was expressed in CAFs, and LH2 depletion abrogated the ability of CAFs to promote tumor cell invasion and migration. Stromal activation of CAFs by tumor cells leading to the enhancement of tumor cell phenotype is the classic example of the role of tumor cells activating CAF with the support of ECM protein in favor of migration and invasion. Yoshida et al. reported a functional link between stromal integrin and CAF activation, initiated by tumor cells, which finally affects migration and invasion of the same tumor cells [51]. The involvement of matricellular glycoprotein, SPARC, has been identified as a part of endometrial tumor cells affecting stromal CAFs in favor of enhancing their phenotypes. The study demonstrated that in the presence of fibronectin, SPARC (secreted from SPARC-expressing endometrial tumor cells) activated fibroblasts. SPARC and fibronectin cooperatively mediate this fibroblast activation to influence mobility and invasion of tumor cells [51].

### 8.2. EMT

Among influencing many metastasis-associated phenotypes, CAF participates in orchestrating EMT in endometrial cancers, commonly via secreted cytokines. Other reported mechanisms by which CAFs induced EMT in endometrial cancer cells have been reported involving the pituitary tumor transforming gene [52]. CAF, in this study, induced pituitary tumor transforming gene over-expression and increased tumor cell invasion and migration in vitro. From the standpoint of EMT, an interesting observation was reported demonstrating a parallel existence of TGF-beta1-S1004A signals in both tumor cells and CAF in endometrial cancers, indicating that endometrial cancer cell invasion and fibrosis share common molecular mechanisms [53]. TGF-beta1 pathways have been implicated in EMT in various epithelial tumor types [54,55,56]. S100 calcium-binding protein A4 (S100A4), also known as fibroblast-specific protein 1 (FSP1), a target of TGF-beta1, mediates the invasion of endometrial tumor cells and EMT [53,57]. S100A4 is also a molecular marker of fibrosis [58] and also present in CAFs [8], although its expression in fibroblasts is strongly variable between different CAF subpopulations [59]. Using conditioned media from fibroblasts isolated and cultured from normal and tumor tissues of the endometrium, Wang et al. demonstrated that CAF induced EMT, migration, invasion, and lung metastasis in endometrial cancer cells through the secreted cytokines. The conditioned media of CAFs decreased E-cadherin levels and increased the levels of N-cadherin and vimentin, which were associated with increasing the levels of invasion and metastasis in tumor cells [60]. As more reports on the mechanism of CAF-tumor cell cross-talk enrich our understanding in endometrial cancers, it becomes apparent that tumor cells are the dominant partners of this cross-talk that orchestrates several tumor cells phenotypes via CAF signaling.

### 8.3. Migration, Invasion, and Metastatic Progression

In endometrial cancers, the major metastasis-associated phenotypes linked with the cross-talk between stromal CAFs and tumor cells are migration, invasion, and metastasis. The mode of cell-to-cell communication is primarily paracrine, as reported using conditioned media or via exosomes, while the intracellular signaling is mediated chiefly through a post-transcriptional gene regulation, miRNA. The role of miRNA has been established in post-transcriptional gene regulation and can function as oncogenes or tumor suppressors in different organ type cancers [61]. A microRNA signature in CAFs from human endometrial cancer has been reported to be implicated in various stages of tumorigenesis [62]. Several miRNAs have been reported to be epigenetically downregulated in endometrial CAFs. Mechanistically, the epigenetic downregulation of a miRNA initiated a cascade of cellular function in CAF, leading to the alteration of soluble secretory signals (chemoattractants/exosomes), which controls neighboring tumor cells phenotypes in a paracrine manner. The downregulation of miR-31 in CAFs has been associated with a corresponding increase in expression of the homeobox gene SATB2 in the context of tumor cell migration and invasiveness in endometrial cancers [62,63]. An exosomal NEAT1 from CAFs has been reported to contribute to endometrial cancer progression via miR-26a/b-5p-mediated STAT3/YKL-40 pathway [64]. A direct transfer of CAF-secreted exosomal miR-320a to endometrial cancer cells has been demonstrated to inhibit their proliferation via downregulation of HIF1alpha, which also led to lowered VEGFA expression in vitro [65].

The Wnt-beta-catenin pathway has been implicated in the tumorigenesis of endometrial cancers [66]. Interestingly, the Wnt pathway has been implicated in the cross-talk between CAF and tumor cells in endometrial tumors. The involvement of miRNA-148a in CAF has been reported to control the migration of endometrial tumor cell lines in vitro via activation of the Wnt-beta-catenin pathway establishing a conditioned media-dependent paracrine signaling between CAF and tumor cells [67]. The miRNA-148a has been identified as a metastatic marker in different tumors [68,69]. Aprelikova et al. reported a DNA methylation-dependent downregulation of miRNA-148a in CAF derived from endometrial tumors compared to matched normal tissue fibroblasts. Cancer-associated stromal myofibroblasts exhibited altered methylation status (Global hypo-methylation) as reported in previous studies [70,71]. Aprelikova et al. confirmed the activation of the Wnt pathway in CAFs and that target genes for miRNA-148a, WNT1 and WNT10B, are secreted. Experiments with conditioned media demonstrated how CAFs overexpression of miR-148a significantly impaired the migration of endometrial cancer cell lines without affecting their growth, while WNT10B stimulated migration [67].

A similar increase in cell invasion and cancer metastasis has been attributed to the endometrial CAFs by an entirely different mode of communication between the CAFs and the tumor cells. Li et al. found that exosomal loss of miR-148b from CAFs induced EMT in endometrial cancer cells as a result of relieving the suppression of DNMT1 [72]. The miR-148b was found to be transferred from CAFs to endometrial cancer cells through exosomes. Their study can be a strong indication that a cross-talk exists between tumor stroma (CAF) and tumor cells which can be functionally relevant to the progression of the disease in endometrial cancers and might be important in determining the prognosis of the disease. 

Apart from miRNA, another cellular signaling axis was reported to mediate the cross-talk between CAF and tumor cells. In all subtypes of endometrial cancers (endometrioid and non-endometrioid tumors), the activation of the PI3K/AKT pathway is a common mechanism in contributing to epithelial-mesenchymal transition (EMT) and cancer stem cell (CSC) features [73]. Tend et al. reported that conditioned media of CAF from endometrioid adenocarcinomas stimulated proliferation, migration, invasion, and induced in vivo tumorigenesis of admixed endometrial cancer cells as compared to the stromal fibroblasts isolated from normal endometrial tissues [74]. Their data indicated that tumor progression was associated with the SDF-1alpha/CXCR4 axis in an autocrine manner, which activated the downstream PI3K/AKT and MAPK/ERK signalings. One of the findings of this study was a positive correlation between high SDF-1alpha expression in 202 patients with myometrial invasion and lymph node metastasis. In the high SDF-1alpha expression group, 23% of patients died and 18% of patients. In sum, the cross-talk once established between CAF and tumor cells directly impacts the clinical parameters of the disease and thus influences the management of the disease and its clinical outcome.

## 9. CAF Influencing Immune-Defence of the Host and Immune-Surveillance of Tumor Cells by the Host in Endometrial Cancers

CAFs are important regulators of the immunological microenvironment of solid tumors, which functions via cytokines and cell-to-cell attachment. In many solid tumors, CAF thus contributes to the resistance to immune therapy [75]. Although the literature on immune reprogramming by CAF is readily available in other solid tumors like PDAC, the study in endometrial cancer has been limited so far. However, a recent interest in this aspect of the study has been fueled by the encouraging results of immune therapy in endometrial cancers, leading to two FDA approvals. In September 2019, FDA approved Pembrolizumab/Lenvatinib (multi-kinase inhibitor including VEGFR/FGFR and others) for advanced endometrial carcinoma who have disease progression following prior systemic therapy (https://bit.ly/2kqmAGq, accessed on 17 September 2019). The approval was supported by findings from the single-arm, multicenter, open-label, multi-cohort phase Ib/II Study 111/KEYNOTE-146 trial (*NCT02501096*), which evaluated 108 patients with previously treated metastatic endometrial cancer treated with Lenvatinib and pembrolizumab. A randomized phase II study demonstrated that Cabozantinib (a multi-kinase inhibitor) plus nivolumab presents improved PFS (Progression Free Survival) compared to nivolumab in heavily pretreated women with recurrent endometrial cancers (*NCT03367741)*. On April 22 2021, the Food and Drug Administration granted accelerated approval to dostarlimab-gxly (Jemperli, GlaxoSmithKline LLC) for adult patients with mismatch repair deficient (dMMR) recurrent or advanced endometrial cancer, as determined by an FDA-approved test, that has progressed on or following a prior platinum-containing regimen. JEMPERLI is a programmed death receptor-1 (PD-1) blocking antibody. Clinical trials using anti-PD-1 or anti-PD-L1 antibodies, but not anti-PD-L2, are currently in, for all types of gynecological cancers. Although the data shows good safety profiles in a certain cohort of patients, yet response rates remain low and many aspects remain controversial [76]. There are several ongoing immunotherapy clinical trials for patients with endometrial cancers (NCT02630823, NCT02725489, NCT02728830, NCT02646748, NCT02914470, NCT02521844). As more immune therapy drugs are FDA approved in uterine cancers, the understanding of the role of CAF in the development of drug resistance to the therapy as well as stromal-normalization in overcoming the drug resistance becomes pertinent. 

CAF has been reported to interact directly with the adjacent tumor cells and the rest of the immune components of TME and indirectly support tumor cells by influencing the host’s immune defense and immune surveillance in solid tumors via the expression of PD-L1 [75,77]. CAFs can also form a nexus with immune cells of the TME, leading to adaptive immune resistance and reducing the host’s immune defense by (1) facilitating CD8+ T-cells exhaustion and/or (2) dampening the priming of CD8+ T-cells. CAFs in conjunction with macrophage type 2 and regulatory T cells (Tregs) have been reported to mediate immunologic barriers against CD8 + T cell-mediated anti-tumor immune responses or simply causing CD8+ T-cell exclusion from tumors leading to adaptive immune resistance [78,79].

CAFs have been demonstrated to perform immune reprogramming in endometrial tumors by suppressing Natural Killer (NK) cells via cell-to-cell interaction. NK cells are a significant component of the host’s immune defense system against tumor cells as they perform immune surveillance in solid tumors, including endometrial cancers. CAF’s ability to suppress the NK cell activity directly helps tumor cells in immune evasion and indirectly helps in the growth and metastasis of cancer [80]. CAFs suppressed the cell-killing activity of NK cells via cell-to-cell interaction; cell surface ligands expressed on CAF interact and inhibit activating cell surface receptors on NK cells. CAF, isolated from endometrial tissue by Inoue et al., suppressed the activity of NK cells via receptor-ligand interactions between CAFs and NK cells. A decrease of cell-surface poliovirus receptor (PVR/CD155), a ligand of activating NK cell receptor DNAM-1, was observed in CAF. The study demonstrated that cell-surface expression of PVR/CD155 is decreased in CAFs compared to normal fibroblasts. Interestingly, the predictive value of PVR/CD155 has been reported in solid tumors. Qu et al. reported that a loss of CD155 expression predicted poor prognosis in hepatocellular carcinoma [81]. 

## 10. Epilogue

Here we presented the functional characteristics of CAF in the context of their origin. We reviewed the nature of CAF’s involvement in regulating growth and several phenotypes of tumor cells involving different oncogenic pathways. Thus, we interrogated the functional characteristics of CAF in the light of its dialogue with tumor cells and other components of TME towards developing a precision strategy of CAF-based therapy in solid tumors with particular reference to endometrial cancers.

A critical review of the subject at hand reveals two apparent facts in the context of CAF-tumor cell cross-talk in endometrial cancers. First, the literature for CAF is limited in endometrial cancers. Although a significant body of literature exists on the function of CAF and their deterministic role in shaping the disease progression in different solid tumors, like PDAC, breast cancers, lung cancers, colorectal cancers, and prostate cancers, the literature is limited for gynecological cancers, especially endometrial cancers. The insufficiency of data can be partly attributed to the impossibility of obtaining longitudinal sampling of the same lesion throughout the disease progression or during treatment to study a real-time conversion between two states of CAF, benevolent and malevolent, as well as their co-evolution with the tumor cells. The scope of repeat biopsy remains limited in the endometrial tumors, as so in many other cases of solid tumors. Second, considering the undeniable role of CAF in progression and drug resistance, the two most critical deterministic factors in the clinical management of a disease, it is puzzling why a CAF-based therapy has not evolved even in other solid tumors, where much more information exists. 

That brings us to the current state of the puzzle in CAF research. Literature from 1966–2021 favors the school of thought that CAFs are benevolent in suppressing the development of cancers [82,83]. It was demonstrated 50 years ago that normal fibroblasts inhibit the growth of polyoma virus-transformed cells [82]. Thus, it is argued that during the initial phase of tumorigenesis, the CAF, in its presumably inactivated state, may not be supportive to the growth of tumor cells. Conspicuously, CAF in its activated form is not the same in effecting tumor growth and progression, keeping the debate in favor or against CAF (good-CAF versus bad-CAF) inconclusive and wide open even in the most studied solid tumors, wherein the contribution of stromal CAF is undeniable, including PDAC and breast cancers [3,4,14,22,84,85,86]. The malevolent transformation of CAF later is believed to be mediated through a number of tumor cell-initiated factors, immunogenic factors, and the physicochemical properties of stroma. In order to conclusively establish CAF as a target for a treatment, we need to establish definitive markers for good-CAF, which are mutually exclusive from the markers of bad-CAF, representing two distinct populations contextually connected to distinct clinical outcomes. To this end, a report by Mizutani et al. identified a Meflin-positive CAF in PDAC, which represented a cancer-restraining population. Their data suggested that Meflin is a marker of cancer-restraining CAFs that suppress progression in PDAC [87,88]. Future studies may identify the presence of such markers in solid tumors, including endometrial cancers, and confirm their role in targeting such markers towards managing the disease.

From the interrogation of the current literature on the ambiguity of CAF, two relevant features surface. There are two characteristics of CAF that we know for certain. First is the heterogeneity in the CAF world, their origin, marker(s), and subpopulation(s). Second is their undeniable role in initiating and the evolution of tumor cells that ultimately determine the disease’s outcome. The first one restricts us from targeting CAF to achieve a successful CAF-based therapeutic strategy. The second one prohibits us from denying the scope of the CAF-based therapeutic approach towards managing the disease. The fact that a number of cross-talks exist between tumor cells and CAF in endometrial cancers proves the importance of the intra-tumoral CAF-tumor cells ecosystem. Furthermore, it explains why and how CAFs co-evolve with tumor cells during the process of metastatic progression of the disease and/or during the development of drug resistance following a clinical intervention, the two most critical determinants of the disease outcome. The only way out of this puzzle is to acquire more knowledge about the heterogeneity and function of CAF, to establish a CAF-based stromal-switch, and to address the dynamic contribution of CAF in the progression of cancer. As we begin an in-depth characterization of CAF and CAF’s functional choreograph with endometrial tumor cells as well as other stromal cells within the uterine TME, studies will unearth novel therapeutic targets [23]. Future work will pave the way to stratify the approach to “normalize” the stromal switch by targeting CAF in endometrial cancers.

## Figures and Tables

**Table 1:** The table presents CAF-Tumor cell cross-talk in endometrial cancers with specific reference to the language and the topic of the cross-talk. The information has been categorized according to the outcome of the cross-talk, including growth, proliferation, stromagenesis MA-phenotypes, EMT, progression, stemness, metabolic reprogramming, and immunological reprogramming.

CAF-Tumor Cell Cross-Talk	Language of Cross-Talk (Mode of Signal Transduction between CAF and Tumor Cell)	Agenda of Cross-Talk (How Cross-Talk Alter Tumor Cell Functions?)	Result of Cross-Talk (Which Tumor Phenotypes Are Affected by Cross-Talk?)	Reference (PMID)
**Growth**				
Angiogenesis focused signals	TGFA, TGFB2 and TGFBR1 and VEGFC	Steroid Hormone-driven proliferation	Growth	25976290
Estrogen increased cHa-ras expression and tyrosine kinase (TK) activity in fibroblast	Induction of c-Ha-RAS transcripts in endometrial cancers; persistent activation of TK led to overexpression of c-Ha-RAS in some endometrial cancer cells under predominant estrogen milieu	Estrogen-driven	Growth	7577718
Estrogen induces expression of c-fos and c-jun in endometrial cancer cell line and fibroblasts	via activation of protein kinase C	Estrogen-driven	Growth	8701784
**Proliferation**				
STAT-3 target genes	Interleukin-6/STAT-3/c-Myc pathway	Non-steroidal	Proliferation	27186396
CAFs secrete higher levels of macrophage chemoattractant protein (MCP)-1, interleukin (IL)-6, IL-8, RANTES, and vascular endothelial growth factor (VEGF)	CAF secretome induced cell proliferation	PI3K/AKT and MAPK/ERK signaling	Proliferation	23922669
**Stromagenesis, MA-Phenotypes, EMT, Progression & Stemness**				
CAF induces gene expression	Pituitary tumor transforming gene in tumor cells	Invasion, Migration, and EMT	MA-Phenotypes	30961403
SPARC-expressing endometrial cancer cells	SPARC from tumor cells activated fibroblasts in the presence of fibronectin	A matricellular glycoprotein, SPARC	Stromagenesis, mobility and invasion of cancer cells	33579227
Exosome-mediated transfer of miR-148b from CAF to tumor cells	Downregulated miR-148b in CAF induced EMT of cancer cell as a result of relieving the suppression of DNMT1	miR-148b as a tumor suppressor by binding to its downstream target gene, DNMT1	EMT	30146796
MicroRNA and transcriptional regulators	SATB2 gene	miR-31	Migration & Invasion	20980827; 21088483
SDF-1α is a novel independent poor prognostic factor	Paracrine- or autocrine- activation of the PI3K/AKT and MAPK/ERK signalings	SDF1alpha /CXCR4 axis	Progression	26851944
Invasive myofibroblasts adjacent to malignant epithelial cells of endometrial cancers showed frequently intensive positive staining of VEGF, IGF1, and EGF, the cognate receptors such as Fetal liver kinase-1/Kinase Insert Domain-containing receptor/VEGF receptor-2, fms-like tyrosine kinase-1/VEGF receptor-1, and EGRF, several cell cycle regulators such as cyclins and cyclin- dependent kinases, and estrogen receptor alpha	Growth factors, their cognate receptors, and HIF-1alpha	Myofibroblasts, as well as cancer epithelial cells, are positive staining for PCNA and Ki-67	Progression	11595701
Activation of the WNT/β-catenin pathway in CAFs	miR-148a expression is suppressed in CAFs; Silencing of miR-148a in CAFs promotes the migration of endometrial cancer cells by targeting Wnt10B to activate the Wnt/β-catenin pathway	WNT10B stimulated migration of endometrial cancer cell lines; WNT10B is a direct target of miR-148a in endometrial CAFs	Cell motility & Invasion	22890324
**Metabolic Reprogramming**				
ROS, produced by CAFs or tumor cells; CAFs-derived exosomes	CAFs exhibit the Warburg effect and activation of the autophagic pathway	A metabolic symbiosis between epithelial cancer cells and CAFs	Metabolic Reprogramming in tumors	26445347
**Immunological Reprogramming**				
Suppression of NK cell activity by CAFs	Cell-to-cell interaction required CAF-induced decrease in NK cell activity	Exosome-independent	Immunological Reprogramming	27499237

## Data Availability

Not applicable.

## Abbreviation

CAF	Cancer-Associated Fibroblast
TGFA/B	Transforming Growth FactorA/B
TK	Tyrosine Kinase
STAT3	Signal transducer and activator of transcription 3
SPARC	Secreted Protein Acidic And Cysteine Rich
SATB2	SATB Homeobox 2
HIF-1alpha	Hypoxia Inducible Factor 1 Subunit Alpha
NK Cells	Natural Killer Cells
DNMT1	DNA Methyltransferase 1
SDF1	Stromal Cell-derived factor 1
CXCR4	C-X-C Motif Chemokine Receptor 4
PCNA	Proliferating Cell Nuclear Antigen
EMT	Epithelial-Mesenchymal Transition
CAF	Cancer-Associated Fibroblast
TGFA/B	Transforming Growth FactorA/B
VEGFC	Vascular endothelial growth factorC
TK	Tyrosine Kinase
STAT3	Signal transducer and activator of transcription 3
SPARC	Secreted Protein Acidic And Cysteine Rich
SATB2	SATB Homeobox 2
HIF-1alpha	Hypoxia Inducible Factor 1 Subunit Alpha
NK Cells	Natural Killer Cells
DNMT1	DNA Methyltransferase 1
SDF1	Stromal Cell-derived factor 1
CXCR4	C-X-C Motif Chemokine Receptor 4
PCNA	Proliferating Cell Nuclear Antigen
EMT	Epithelial-Mesenchymal Transition
CAF	Cancer-Associated Fibroblast
NK Cells	Natural Killer Cells
MDSC	Myeloid deriver stromal cells
TME	Tumor microenvironment
